# The Level of Cadmium Impurities in Traditional Herbal Medicinal Products with *Plantago lanceolata* L., folium (Ribwort Plantain Leaves) Available in Polish Pharmacies — Comprehensive Toxicological Risk Assessment Including Regulatory Point of View and ICH Q3D Elemental Impurities Guideline

**DOI:** 10.1007/s12011-021-02861-5

**Published:** 2021-08-10

**Authors:** Kamil Jurowski, Maria Fołta, Barbara Tatar, Mirosław Krośniak

**Affiliations:** 1 Institute of Medical Studies, Medical College, Rzeszów University, Al. mjr. W. Kopisto 2a, 35-959 Rzeszów, Poland; 2 Department of Food Chemistry and Nutrition, Medical College, Jagiellonian University, Medyczna 9, 30-688 Kraków, Poland

**Keywords:** Plantago lanceolata L., folium (ribwort plantain), Cadmium, ICH Q3D, Toxicology, Elemental impurities

## Abstract

*Plantago lanceolata* L., folium (ribwort plantain) is an active pharmaceutical ingredient in traditional herbal medicinal products (THMP) available in pharmacies as a demulcent for the symptomatic treatment of oral or pharyngeal irritations and associated dry cough. This kind of THMP is widely applied among the European population; however, recently, voices have been heard in the public about alleged cadmium impurities. The idea of our study was a comprehensive risk assessment of the Cd impurity exposure and its effect on human health. Our assessment strategy was based on regulatory aspects (raw results versus limits set by FAO/WHO for herbal medicines) and toxicological risk assessment approach based on ICH Q3D guideline for elemental impurities in final pharmaceutical products. The cadmium was determined by electrothermal atomization atomic absorption spectrometry based on our previously described methodology. Obtained results show that all analysed THMP with *P. lanceolata* L., folium available in the Polish pharmacies contain cadmium impurities, but at a very low level (ranged 0.73 – 20.6 μg/L). The content of Cd in a single dose (ng/single dose) is also very low and is not a threat to patients. The estimated maximum daily exposure (ng/day) of Cd based on the ‘worst-case scenario’ (maximum concentrations by oral administration) meets the standards of ICH Q3D guideline (all results were below oral permitted daily exposure; PDE for Cd, i.e. < 5.0 µg/day). It can be concluded that all analysed samples of THMP with *P. lanceolata* L., folium should not represent any health hazard to the patients due to cadmium levels. To the best of our knowledge, this is the first study about Cd impurity level in THMP with *P. lanceolata* L., folium (ribwort plantain leaves) available in European pharmacies.

## Introduction

Herbal medicinal products (HMP) usually contain different herbal substances as an active pharmaceutical ingredient (API) [1]. Out of all European Union herbal monographs (*n* = 165; July 2021) [2], a very intriguing and interesting herbal plant applied in the European pharmaceutical industry is *Plantago lanceolata* L., folium (ribwort plantain). The pharmaceutical products with *P. lanceolata* available in pharmacies are well known and usually applied (especially by population in Europe) as a demulcent for the symptomatic treatment of irritations of oral and pharyngeal mucosa with associated dry cough [3]. *P. lanceolata* L. is a species of the genus *Plantago* in the Plantaginaceae botanical family [3]. This plant is a common perennial weed of arable fields and grassland [4], ubiquitous across Europe and North and Central Asia [5]. Based on the scientific literature review from the last few years, many studies including different element concentrations in this plant also contaminated sites [6–10]. Especially interesting were studies described by Nadgórksa-Sochna et al. [11] about the concentration of Cd (1.0 – 13.8 mg/kg d.w.) in the leaves of P. *lanceolata* during the flowering stage from different geographical regions in Poland. Additionally, important studies about applications of P. *lanceolata* in environmental pollution research in an urban area of Southern Poland were described by Skrynetska et al. [12]. The aim of the mentioned article was analysis of the Cd content (and other elements) in the leaves (washed/unwashed) of *P*. *lanceolata* in different kinds of urban areas: road (7.9 mg/kg, washed leaves; 10.0 mg/kg, unwashed leaves), park (8.4 mg/kg, washed leaves; 10.4 mg/kg, unwashed leaves), and metallurgical plant (8.0 mg/kg, washed leaves; 9.9 mg/kg, unwashed leaves). Obviously, people are not collecting this plant for consumption in polluted areas; however, aforementioned studies justify conducting research in relation to final pharmaceutical products manufactured in Poland. It is important because monitoring of elemental impurities (EI) in final traditional HMP (THMP) based on ICH Q3D elemental impurity guideline published by EMA [13] is highly desired and is still a relevant regulatory problem. However, there is very little attention about this topic among scientists. The justification of our studies is the fact that monitoring of heavy metals impurities (like Cd) in final pharmaceutical products should be mandatory due to ICH Q3D elemental impurity guideline. Therefore, the aim of our article was a comprehensive toxicological risk assessment of cadmium in THMP with *Plantago lanceolata* L., folium (ribwort plantain leaves) based on ICH Q3D guideline. The choice of this metal was justified by its potential possibility of occurrence in this herb (based on mentioned earlier literature review) and also our apparatus capabilities. From the toxicological point of view, this topic is extremely important because oral exposure of Cd can be very harmful for human health. It should be mentioned that a sensitive endpoint for oral exposure to Cd and its compounds is renal toxicity [14]. Additionally, skeletal and renal effects are observed at similar exposure levels and are a sensitive marker of this element exposure [15]. On the other hand, several oral exposure studies of this element in rats and mice showed no evidence of carcinogenicity. Therefore, the renal toxicity endpoint was crucial to establish the oral permitted daily exposure (PDE) for cadmium. Due to the lack of knowledge about cadmium impurities in THMP with *Plantago lanceolata* L., folium, this article is focused on comprehensive toxicological risk assessment including a regulatory point of view and the ICH Q3D elemental impurity guideline.

## Materials and Methods

### Samples

In Poland, various herbal products containing *Plantago lanceolata* with a traditional indication are available on the market. None of them, however, fulfils the requirement of a medicinal use [3]; hence, in our studies, we investigated THMP with *P. lanceolata* L., folium (ribwort plantain leaves) available only in Polish pharmacies (*n* = 5). The choice of investigated THMP was justified by availability in Polish pharmacies as a representative example of a European country. Additionally, literature overview indicated a lack of studies related to final THMP with this plant. All pharmaceutical products with *P. lanceolata* L. were purchased in local pharmacies situated in Poland (Kraków, Niepołomice, Rzeszów) in 2021 (in period: March – April). All purchased THMP were pharmaceutical products classified as over-the-counter medicines of individual manufacturers. It should be underlined there are only five manufacturers of these products in Poland. A brief description of the investigated THMP is presented in Table 1.

**Table 1:** The brief description of the analysed traditional herbal medicinal products with *Plantago lanceolata* L., folium available in Polish pharmacies

Sample		Herbal plant	Traditional herbal medicinal product	Preparation	Posology	License	Lot number	Note
No	Code							
1	A	*Plantago lanceolata* leaves	*Plantaginis lanceolatae folii extractum fluidum*	Liquid extract (1:2–2.5) extraction solvent ethanol 60% (V/V)	Oral use: 7.5 –15 mL (1.125–2.25 g of extract) 4–5 times daily	10,082	2,072,019	OTC
2	B	*Plantago lanceolata* leaves	*Plantaginis lanceolatae folii extractum fluidum*	Liquid extract (1:1–2) extraction solvent ethanol 30% (V/V)	Oral use: 5 mL 3–4 times daily or 10 mL 2 times daily (100 g syrup contains 10 g of extract)	R/2954	60,520	OTC
3	C	*Plantago lanceolata* leaves	*Plantaginis lanceolatae folii extractum fluidum*	Extract (1:7) extraction solvent ethanol/water (95:5)	Oral use: 5–10 mL (2.17–4.34 g of extract) 3–4 times daily	22,350	70,220	OTC
4	D	*Plantago lanceolata* leaves	*Plantaginis lanceolatae folii extractum fluidum*	Liquid extract (0.7–1.3:1) extraction solvent ethanol 20% (m/m)	Oral use: 6.4–19.2 g (0.32–0.96 g of extract) 2–5 times daily	9981	200,202	OTC
5	E	*Plantago lanceolata* leaves	*Plantaginis lanceolatae folii extractum fluidum*	Liquid extract (1:3) extraction solvent ethanol 60%	Oral use: 5–15 mL (0.647–1.941 g of extract) 3–4 times daily	9021	NA	OTC

For the best quality of methodological standards, the double-blind approach was applied. For this purpose, all samples were coded in random order (A, B, and so on). To minimize any potential impurities (also elemental) from other sources, all the steps during the sampling procedure were carried out in analytical and clinical purity in the Bioelements Laboratory at Department of Pharmacy, Collegium Medicum, Jagiellonian University, in Kraków. Additionally, to avoid any impurities during studies, the plastic equipment was applied. Moreover, laboratory glassware (volumetric flasks, funnels, etc.) were kept overnight in a 10% nitric acid (HNO_3_) solution and rinsed with deionised water and air dried before analysis. It should be noted that pre-treatment and treatment steps of samples (homogenization and digestion in nitric acid) were unnecessary because all samples were in liquid form (drops). Hence, in situ analysis was applied at the measurement step (see “Instrumentations and Determination of Cadmium” section).

### Chemicals

All reagents applied were of analytical grade and for the preparation of all solutions demineralized water (Millipore) was applied. Ultrapure demineralized water had been obtained by Milli-Q water purification system (Millipore, Bedford, MA, USA).

Nitric acid (65%) (for standard solutions) was of spectroscopic grade (Merck SupraPur, Darmstadt, Germany). Standard solutions of cadmium (Cd standard solution traceable to SRM from NIST – Cd(NO_3_)_2_ in 0.5 mol·L^−1^ HNO_3_,and 1000 mg L^−1^ Cd (CertiPUR®, catalog product: 1.19777.0500) was prepared by dilution of certified standard solutions (1000 μg·L^−1^ MERC). The certified reference material (BCR-482; IRMM, Belgium) was material prepared from lichen. The utility of this certified reference material in cadmium determination with respect to plant material is justified by the studies described by Coufalík et al. [16]. The purge gas was argon at purity 99.99%.

### Instrumentations and Determination of Cadmium

The determination of Cd in the investigated samples was carried out using the Perkin-Elmer 5100 ZL atomic absorption spectrometer (Perkin-Elmer, Norwalk, CT, USA) with Zeeman background correction and with electrothermal atomization (ETAAS technique) using the appropriate hollow cathode lamp. Pyrolytically coated graphite tubes with L’vov platforms were applied. The appropriate design time–temperature programme for Cd determination was developed. The injection volume was 20 μL and integrated absorbance (peak area) was applied for signal evaluation. The description of instrumental and experimental conditions for ET-AAS determinations of Cd are shown in Table 2.

**Table 2:** Description of instrumental and experimental conditions for ET-AAS determinations of Cd

Instrumental/experimental condition(s)	Value for Cd determination
Wavelength, nm	228.8
Lamp current, mA	5.0
Optimum working range (µg/kg)	0.02 – 0.20
Slit, nm	0.7
Step 1, °C	120
Ramp/Hold, s	10/25
Step 2, °C	300
Gas flow (mL·min^−1^)	250
Ramp/hold, s	5/15
Gas flow (mL·min^−1^)	250
Step 3, °C	1600
Ramp/hold, s	0/3
Gas flow (mL·min^−1^)	0
Step 4, °C	2400
Ramp/hold, s	1/2
Gas flow (mL·min^−1^)	250
L’vov platform	Yes
Integration time, s	4
Injection volume, µL	20.0

### The Toxicological Risk Assessment of Cd Impurities Due to ICH Q3D Guideline

**Fig. 1:**
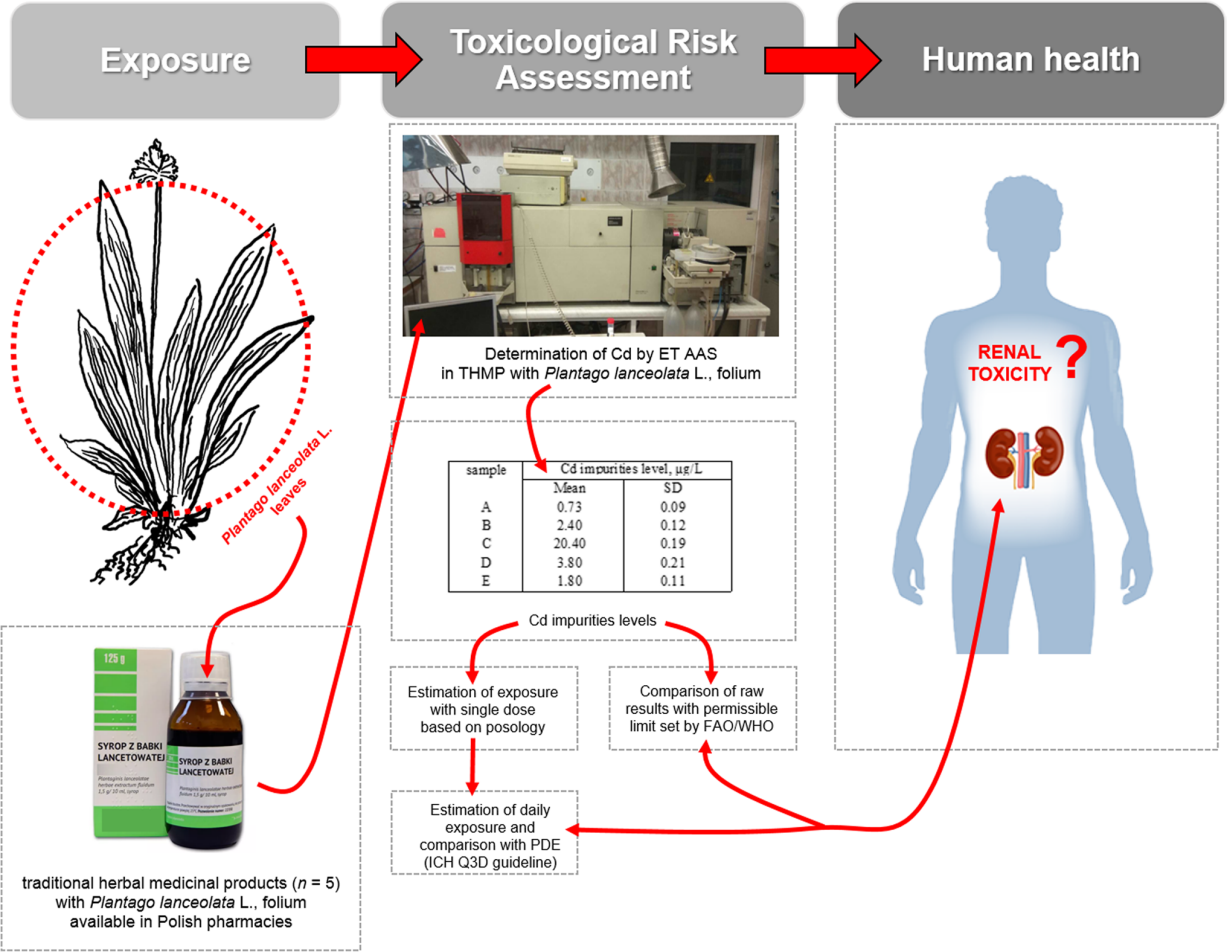
The workflow of the applied toxicological risk assessment of cadmium in THMP with *Plantago lanceolata* L., folium (ribwort plantain leaves) available in Polish pharmacies

The appropriate toxicological risk assessment (TRA) of heavy metals impurities usually is based on only determination of each heavy metal in analysed samples. However, because investigated samples were final THMP available in pharmacies, the applied TRA procedure of Cd determination was in situ analysis with appropriate designed toxicological risk assessment approach. The workflow of our study is graphically presented in Fig. 1.

### Analytical Calibration and Analytical Quality Control Strategies

The analytical calibration procedure was performed using single calibration solutions (0.5 mol/L HNO_3_) prepared from 1 g/L Merck p.a. stock solutions as was described in an earlier section. Working solutions (*n* = 6) with concentrations 0.5, 1.0, 2.5, 5.0, 10.0, and 25.0 μg/L were prepared and used for the analytical calibration procedure. The values of the correlation coefficient (*R*) are good indicators of the linearity for AAS instruments giving precise and accurate results. The obtained value of correlation coefficient (*R* = 0.999) indicated that the analysis was precise and accurate. The recovery was calculated (99.6%) as the quotient of the determined level and the known amount of the determined element expressed as a percentage. The calculated limit of detection (LOD) was 0.15 µg/L. The calculated LOQ was 0.34 µg/L.

As was mentioned in the “Chemicals” section, the certified reference material was material prepared from lichen (BCR-482; IRMM, Belgium). Before the analysis, lichen samples were dried at 70 °C for 24 h. After drying, the samples were transferred into solution by microwave digestion (digestion was carried out by means of programmable microwave oven model MDS-2000; CEM Corp., Matthews, USA). First, 5 mL of nitric acid (65%) was added to 300 mg of the sample of certified reference material in Teflon reaction vessels and left to pre-digest for 24 h. Then, digestion was carried out, and after cooling the vessels, the contents were quantitatively transferred to Sarstedt vessels and completed with demineralized water to a total volume of 15 mL. Samples prepared in this way were analysed using a Perkin-Elmer 5100 ZL atomic absorption spectrometer in graphite furnace mode (as analysed samples of THMP). The target value of Cd was 0.560 ± 0.020 mg/kg and the obtained value was 0.528 ± 0.011 mg/kg. Additionally, the quality control and validation of applied methodology were confirmed by our previously described studies using the similar methodology and apparatus [17–19].

### Statistical Analysis

The obtained results were analysed using statistical two software: (1) Excel 2010 (Microsoft Office) and (2) Origin 2021 Pro the Ultimate Software for Graphing and Analysis (OriginLab Corporation, One Roundhouse Plaza, Suite 303, Northampton, MA 01,060, USA) licensed by the Jagiellonian University in Krakow. Data processing, all basic descriptive calculations, compilation, and storage of obtained data at laboratory stage were performed using Excel 2010 (Microsoft Office). The obtained results were analysed applying Origin 2021 Pro (initial analysis, not included). The results of five independent replicates (five determinations) for each sample (*n* = 5) were expressed as the mean ± standard deviation. Additionally, the descriptive statistics were made (minimum, maximum, mean, standard deviations, skewness, and kurtosis) using Origin 2021 Pro.

## Results and Discussion

### The Cadmium Impurity Levels of Traditional Herbal Medicinal Products with *Plantago lanceolata* L., folium (ribwort plantain leaves) Available in Polish Pharmacies

The cadmium impurity levels of all investigated THMP samples (*n* = 5; A – E) are shown in Table 3 (μg/L). Additionally, the descriptive statistics of Cd impurity level in investigated samples is briefly summarised in Table 4.

**Table 3:** The cadmium impurity levels in traditional herbal medicinal products (A – E) with *Plantago lanceolata* L., folium (ribwort plantain leaves) available in Polish pharmacies

Sample	Cd impurity level, μg/L	
	Mean	SD
A	0.73	0.09
B	2.40	0.12
C	20.40	0.19
D	3.80	0.21
E	1.80	0.11

*SD* standard deviation

**Table 4:** The descriptive statistics of Cd impurity levels in samples of traditional herbal medicinal products with *Plantago lanceolata* L., folium (ribwort plantain leaves) available in Polish pharmacies

Parameter	Minimum, μg/L	Maximum, μg/L	Mean, μg/L	SD	Skewness	Kurtosis
value	0.73	20.6	5.84	0.09 – 0.19	1.54	2.68

*SD* standard deviation

The overview of the obtained raw results shows the highest level of cadmium in sample C (20.6 ± 0.19 μg/L) and the lowest level in sample A (0.73 ± 0.09 μg/L). In general, the cadmium levels were similar for all analysed THMP (in the range of 0.73 to 3.8 μg/L), except sample C — approximately 30 times higher than the lowest cadmium level in other samples. Skewness and kurtosis values confirm the distribution of results and their consistency.

The relatively high level of Cd in sample C may indicate a serious health risk. From the regulatory point of view, appropriate health risk assessment is based on comparison of raw results in the final pharmaceutical products with the applicable limits. In this situation, it is possible to compare our results with two important sources. The first source is the permissible limit set by FAO/WHO for herbal medicines. The second source is the level limit recommended by the ICH Q3D guideline. The obtained raw results show that all samples contained Cd levels below the permissible limit set by FAO/WHO for herbal medicines (10 mg/kg [20]). What is more, considering the level limits for Cd in pharmaceuticals via the oral route recommended by the ICH Q3D guideline (500 μg/kg [13]), all of the investigated samples meet also the requirements. The obtained results about range of Cd impurities can be useful not only for other researchers but also for regulatory entities.

### The Estimation of Cadmium Oral Exposure for Human Health in Traditional Herbal Medicinal Products with *Plantago lanceolata* L., folium (Ribwort Plantain Leaves) Available in Polish Pharmacies

The comprehensive TRA should include not only the comparison of raw results with appropriate limits (FAO/WHO [20] or ICH Q3D guideline [13]) but additionally an appropriate estimation of oral exposure. In this situation, the first step should be estimation of exposure for a single dose based on posology (Table 1) and obtained raw results from Table 3. Assuming a ‘worst-case scenario’ (maximum concentrations by oral administration) and considering the mentioned earlier data, the exposure to cadmium in the analysed samples is presented in the first line of Table 5 (single dose, ng/single dose). This step is necessary for the final step of assessment of Cd exposure in daily intake of applied THMP.

**Table 5:** The estimated exposure of Cd to which the patient is exposed for single dose (first line of table) and maximal daily dose (second line of table) of the traditional herbal medicinal products with *P. lanceolata* L., folium (ribwort plantain leaves) available in Polish pharmacies

Estimated oral exposure of Cd based on posology	Traditional herbal medicinal products with *P. lanceolata* L., folium (ribwort plantain leaves)				
	A	B	C	D	E
Single dose, ng/single dose	10.95	48.02	204.52	76.32	27.23
Maximum daily dose, ng/day	54.75	192.45	816.12	380.61	108.12

The daily exposure (ng/day) was estimated based on frequency of use (Table 1, posology column) and is presented in Table 5. The obtained results show that the estimated maximum daily exposure of this element is variable among analysed samples (54.75–816.12 ng/day). Because many studies about oral exposure to cadmium in rats and mice showed no evidence of carcinogenicity [14, 15], the main point for TRA was nephrotoxicity [14, 15]. Based on the value of PDE for Cd for oral pharmaceutical products (5.0 μg/day [13]) suggested by the ICH Q3D guideline, all investigated products meet the requirements. What is more, our results indicated that all analysed THMP are characterized by results much lower than PDE value for Cd (about 6 – 90 times lower).

## Conclusions and Recommendations

The justification of our studies is the fact that monitoring of heavy metals impurities (like Cd) in final pharmaceutical products should be mandatory due to the ICH Q3D elemental impurity guideline. The level of Cd impurities in all analysed THMP (A – E) with *Plantago lanceolata* L., folium (ribwort plantain leaves) available in Polish pharmacies occurs but at a very low level (0.73 – 20.6 μg/L). The source of these impurities can result not only from production stages, but also especially from environmental contamination of the *P. lanceolata* L., folium. Content of Cd in a single dose of THMP (ng/single dose) is also very low and is not a threat to patients. The estimated maximum daily exposure (ng/day) of cadmium meets the standards of ICH Q3D elemental impurity guideline (all results were below the established oral PDE for Cd, i.e. < 5.0 µg/day). It can be summarised that our article shows that all analysed THMP with *P. lanceolata* L., folium available in pharmacies in Poland should not represent any health hazard to the patients due to cadmium impurity levels. To the best of our knowledge, this article is the first to study about Cd impurity levels in final THMP with *P. lanceolata* L., folium (ribwort plantain leaves) available in European pharmacies.

It should be underlined that the monitoring of elemental impurities in herbal medicinal products is an extremely rare topic; hence, it would be valuable to carry out a broader study including other elemental impurities to build upon this data like the studies recently described by our group [21–25].

## Data Availability

All data generated or analysed during this study are included in this published article.

## Abbreviations

AAS: Atomic absorption spectrometry technique
API: Active pharmaceutical ingredient,
EI: Elemental impurities
ET AAS: Electrothermal atomization atomic absorption spectrometry
THMP: Traditional herbal medicinal product
ICH Q3D: International Council for Harmonization of Technical Requirements for Pharmaceuticals for Human Use
PDE: Permitted daily exposure
TRA: Toxicological risk assessment
SD: Standard deviation
